# Supplementary material for: Axonal injury is a targetable driver of glioblastoma progression
Source: Nature. 2025 Aug 20;646(8084):452–61. doi: 10.1038/s41586-025-09411-2 (PMC12507684; doi:10.1038/s41586-025-09411-2)
Supplement: Supplementary file 1 — Supplementary Methods for the agent-based modelling framework, related to Extended Data Fig. 7; Supplementary Data Fig. 1 (flow cytometry gating strategy); Supplementary Data 2; and full descriptions for Supplementary Tables 1–10. [file 41586_2025_9411_MOESM1_ESM.pdf]

nature portfolio

https://doi.org/10.1038/s41586-025-09411-2

Supplementary information

Axonal injury is a targetable driver of glioblastoma progression

In the format provided by the authors and unedited

Supplementary methods: Agent-based modelling framework

March 25, 2025

Agent-based model of tumour growth

We developed a stochastic agent-based model of tumour growth to gain mechanistic insights into glioblastoma progression in the striatum[1,2]. The model considers tumour cells to be autonomous agents with their own individual properties. These properties are subject to stochastic effects induced by both the cell population dynamics (*e.g.* cell-to-cell interactions) and the environment (e.g. properties of the white matter and grey matter). The environment where the agents live is assumed to be a 2-dimensional lattice, to which we assign various properties that influence the way agents behave and evolve. This dynamic framework can give rise to highly heterogeneous phenotypes of glioblastoma progression.

Environment

The agents (tumour cells) are assumed to reside and evolve on a 2-dimensional orthogonal lattice. This environment consists of white matter (WM) bundles of myelinated axons, which are surrounded by grey matter (GM), resembling the composition of the striatum. As the anatomy of the striatum varies between individuals, we here assume that WM bundles are equisized ellipses that are uniformly distributed across the GM background. Each site on the orthogonal lattice is assigned with a "white" or "grey" colour, meaning that it lies either within a WM bundle or in GM, respectively. We assume that each lattice site can be occupied by maximum one agent. The space includes in total 200×200 lattice sites, with approximately 20% and 80% of the sites belonging in WM and GM, respectively. We additionally define local neighbourhoods on the lattice. The neighbourhood $\mathcal{N}^r_{x,y}$ of radius r of a site with coordinates (x, y) is

$$\mathcal{N}^r_{x,y} = \{(x', y') : |(x, y) - (x', y')| \leq r\} \tag{1}$$

where $|\cdot|$ denotes Euclidean distance.

Probabilistic rules of evolution

At each time-step, any tumour cell (agent) can either move, divide or die, based on an associated mobility rate p^{mob}, proliferation rate p^{div} and death rate p^{d}, respectively. These rates express the probability per unit time for an event to occur. For a tumour cell to be able to move or divide, it is required that there is a free site in its immediate neighbourhood (with radius $r = 1$). If all neighbouring sites are already occupied (e.g. when the tumour cell is trapped within a tumour bulk) we check whether there are free sites in its neighbourhood of radius $2 \leq r \leq 4$. In that case, the tumour cell is allowed to push its neighbours with probability $p^{\text{push}} = e^{-0.4\,r}$ (that is exponentially decreasing with the radius), in order to move or divide. Additionally, a tumour cell is allowed to diffuse from white to grey matter or vice versa according to a flux rate p^{flux}.

Whenever a tumour cell occupies a site with coordinates (x, y) in the WM, it can induce local injury i with an injury rate p^{inj}. Additionally, we assume that local injury causes a long range inflammation that also contributes to the total injury levels of the neighbourhood of the tumour cell. Therefore, the level of injury on a site with coordinates (x, y) at some timepoint $t > 0$ depends on the accumulated injury in the neighbourhood $\mathcal{N}^{r_{\text{inj}}}_{x,y}$ of radius r_{inj} around (x, y) up to time $t - 1$, as summarised in the following update rule:

$$i_t(x, y) = i_{t-1}(x, y) + \left(\underbrace{c_{\text{inj}}(1 - i_{t-1}(x, y))}_{\text{local injury}} + \underbrace{c_{\text{infl}} \sum_{(x',y') \in \mathcal{N}^r_{x,y}} i_{t-1}(x', y')}_{\text{inflammation effect}} \right) \delta^{\text{inj}}_t(x, y) \tag{2}$$

where

$$\delta^{\text{inj}}_t(x, y) = \begin{cases} 1, & \text{with probability } p^{\text{inj}} \\ 0, & \text{with probability } 1 - p^{\text{inj}} \end{cases} \tag{3}$$

is a Bernoulli random variable indicating whether injury occured on the site (x, y) at time t. The parameters c_{inj} and c_{infl} determine the increase of injury and inflammation levels, respectively. The functional form of the injury ensures that $0 \leq i_t(x, y) \leq 1$ at all times t, so that the injury level saturates after it has reached a certain high level. Tracking the injury $i_t(x, y)$ allows us to model Wallerian degeneration.

In our model, both mobility rate p^{mob} and proliferation rate p^{div} are varying in space and time as they depend on the local injury $i_t(x, y)$. In particular, the mobility rate of a tumour cell that is located at site (x, y) at time t is given by:

$$p_t^{\text{mob}}(x, y) = p_0^{\text{mob}} \frac{1}{1 + c_{\text{mob}}\, i_t(x, y)} \tag{4}$$

where p_0^{mob} is the basal (intrinsic) mobility rate, that is independent of location and c_{mob} is a parameter that controls the sensitivity of tumour cell mobility (diffusion) on injury. Also, the proliferation rate at site (x, y) and time t is given by:

$$p_t^{\text{div}}(x, y) = p_0^{\text{div}} + c_{\text{div}} \frac{1}{1 + \left(\frac{i_{\text{thres}}}{i_t(x, y)}\right)^n} \tag{5}$$

where p_0^{div} is the basal (intrinsic) proliferation rate and the second term is a Hill function of the local injury $i_t(x, y)$: c_{div} is a parameter that controls the increase of proliferation rate as the level of injury surpasses a threshold i_{thres}. We also set the Hill coefficient to be $n = 8$.

Wild type and *Sarm1* mutant scenarios

To simulate both the wild type and *Sarm1* mutant scenarios using the agent-based model we apply the same rules as described in the previous paragraph and only vary some parametric assumptions. We run the models for a total of 100 and 150 timepoints, respectively, corresponding to representative survival days observed for WT and mutant in the experiments.

We make three key assumptions that control how injury accumulates in the WT and *Sarm1* mutant and drives the different phenotypes of tumour growth and progression:

- In the *Sarm1* mutant, we assume that injury starts accumulating only after $t \geq 30$ days, in accordance with experimental observations.
- We assume lower probability of injury in the *Sarm1* mutant compared to the WT.
- We assume that the sensitivity of tumour cell mobility (diffusion) rates on injury is higher in WT compared to the *Sarm1* mutant.

We assume the initial conditions of the simulations to represent a very early stage of tumour progression, starting with $n = 100$ cells placed randomly on the lattice. To match early relative proportions of tumour cells observed in experiments, we start with 60% of cells in WM and 40% of cells in GM for the wild type, and with 50% in WM and GM for the mutant. To compute summary statistics from the model, for each scenario we run in total 5 simulations (replicates), starting with different initial conditions. Then for each summary statistic of interest we compute mean and standard deviation of the statistic over the different replicates. All the parameters used in the simulations of the two scenarios are summarised in Table 1.

Results and discussion

Our aim with this agent-based modelling framework was to reproduce the key qualitative experimental observations in both WT and *Sarm1* mutant scenarios. As basic mechanisms with regards to tumour growth and mobility, and injury signals give rise to emergent behaviours that produce distinct phenotypes, this kind of modelling can shed light into the mechanistic origins of observed phenotypes. Rather than systematic model fitting, we used manual exploration of the model parameters (see Table 1) to generate qualitative behaviour that agrees with experimental observations (Extended data figure 8(b-f)). Snapshots of time evolution sampled from one example simulation run for both WT and *Sarm1* mutant are shown in Extended data figure 8(g), illustrating the faster evolution to bulkier

tumours in the WT, versus a slower development to more diffuse tumours in the mutant. Importantly, the only differences between the WT and *Sarm1* mutant model parameters are a delayed and slower development of injury in *Sarm1*, along with a significant difference in how injury restricts mobility in the WT. We found that the later is crucial for both the retention of tumour cells in WM in the WT and the significantly more disperse tumours in the mutant. Interestingly, the delayed increase in proliferation in the mutant can be explained purely by a delayed and more gradual accumulation of injury, despite the dependence of proliferation rate on injury being the same for WT and mutant. Finally, we observe higher variability in the final tumour population size in the mutant compared to WT, which is also observed experimentally. In summary, our agent-based model supports the conclusions of our paper, demonstrating that all observed phenotypes in the WT and *Sarm1* mutant are effectively captured by assuming that injury induced by tumour cells in WM causes retention as well as rapid tumour proliferation.

Table 1: **Parameter values used in agent-based model simulations.**

**Parameter**	**Wild type**	***Sarm1* mutant**
p_0^{mob}	40.0	40.0
p_0^{div}	0.01	0.01
p^{d}	0.00004	0.00004
p^{flux}	0.1	0.1
p^{inj}	0.5	0.35
r_{inj}	15	15
c_{inj}	0.014	0.014
c_{infl}	0.0035	0.0035
c_{mob}	100	0.1
c_{div}	0.15	0.15
i_{thres}	0.7	0.7

References

1. Jørgensen, A. C. S., Ghosh, A., Sturrock, M. & Shahrezaei, V. Efficient Bayesian inference for stochastic agent-based models. *PLOS Computational Biology* **18,** 1–28 (2022).
2. Jørgensen, A. C. S. *et al.* Data-driven spatio-temporal modelling of glioblastoma. *Royal Society Open Science* **10,** 221444 (2023).

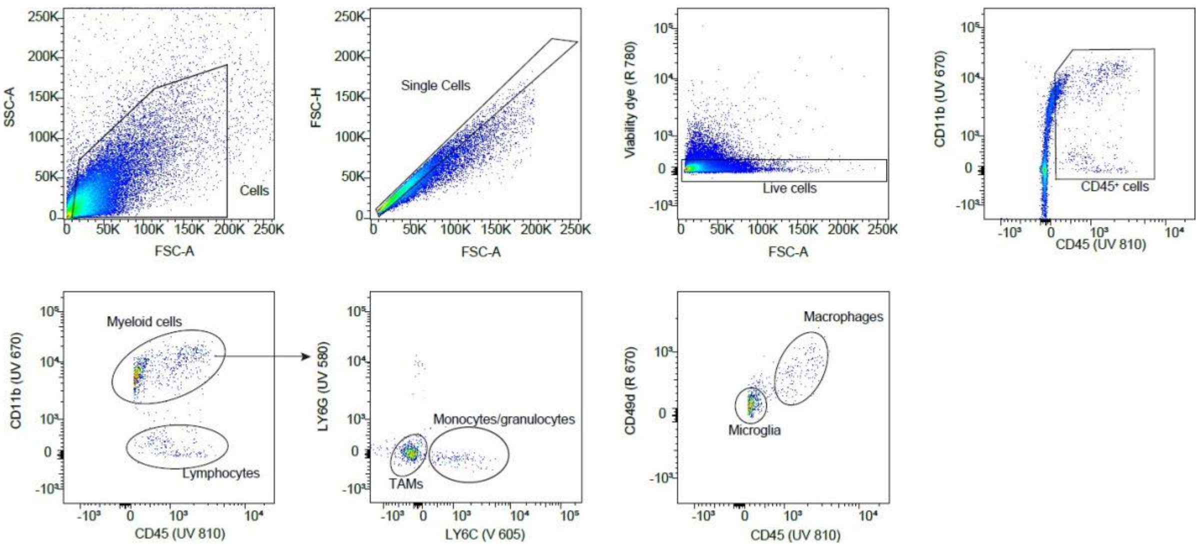

Supplementary Data 1: Gating strategy for flow cytometry experiments in Fig. 5i-m and Extended Data Fig. 4h-l

All cells were first gated in FSC/SSC according to cell size and granularity. This population was then gated in FSC-A/FSC-H to contain only single cells. Next, single cells were gated based on the viability dye, and live cells (negative population) were used for further cell type identification. CD45+ cells were identified in CD45/CD11b. This immune cell population was then gated in CD45/CD11b again to separate myeloid population (CD11b high) and lymphocytic population (CD11b low). The myeloid cells were then further gated in LY6C/LY6G for the double negative population corresponding to tumour associated microglia/macrophages (TAMs). Finally, this population was gated in CD45/CD49d to separate microglia and peripherally derived macrophages (CD45 and CD49d high).

Supplementary Data 2: Description of neuropathology features of terminal tumours formed in WT and *Sarm1*$^{-/-}$ mice, related to Extended Data Fig. 7a

All terminal tumours in WT and *Sarm1*$^{-/-}$ mice replicate the features of human IDH wildtype diffuse astrocytomas, including diffuse infiltration into the surrounding brain with involvement of the corpus callosum, extension to the contralateral hemisphere and cortex. Combined with their molecular profile, tumours in both genotypes model human glioblastoma according to the 5th edition WHO classification (2021).

The majority of tumours in WT (12/15) and minority in *Sarm1*$^{-/-}$ (1/8) animals were composed of densely cellular sheets of severely atypical cells with fibrillary or scanty cytoplasm and intensely hyperchromatic nuclei. Mitotic activity was readily identified and neoplastic cells with large or multiple nuclei were common. A few vessels were lined by hypertrophic endothelium. Focal haemorrhages were common and occasional microfoci of necrosis were also seen.

The remaining tumours in WT (3/15) and the majority in *Sarm1*$^{-/-}$ (7/8) mice showed variable cellularity from low to dense and were composed of cells with fibrillary cytoplasm and mildly to severely atypical nuclei. Atypia was most pronounced in dense regions, which also displayed mitotic activity. Necrosis, microvascular proliferation and haemorrhages were absent. Together, these histological features are indicative of less advanced disease.

Supplementary Table Legends

Supplementary Table 1. Description of patient-derived lines used in this study, related to Fig. 2 and Extended Data Fig. 2
Description of patient-derived lines used within this study, including main mutations and classification of transcriptional profiles according to the Neftel et al and Gangoso et al studies. nd = not determined.

Supplementary Table 2. Processing statistics for Spatial Transcriptomics data, related to Fig. 2 and Extended Data Fig. 2
Output of Space Ranger 2.0.1 for 30 10x Genomics Visium datasets from 14 PDX tumours (10 different cell lines) and one control brain (2 sections per sample). “Sample ID” contains the cell line name followed by 1 or 2 denoting the first and second section respectively. NSG denotes control samples. Columns 6 and 11 to 33 are fractions.

Supplementary Table 3. Meta data for Spatial Transcriptomics experiments, related to Fig. 2 and Extended Data Fig. 2
Annotation of all ST spots reported in this study. Their qualitative tumour contents (“ROI”), Sequencing-based tumour density measure (“gene-based tumour density”), tumour density measured from H&E images (“Image-based tumour density”) and anatomical location in the brain (“Anatomical”) are reported. “CC”: Corpus Callosum; “STR”: Striatum.

Supplementary Table 4. Gene enrichment analysis of gene clusters up-regulated in myelin high spots, related to Fig. 2b
Gene enrichment analysis for 5 clusters (Figure 2b). “GeneRatio”: Ratio of upregulated input genes present in a given GO term over the total number of upregulated input genes; “BgRatio”: Ratio of all genes annotated in a given GO term over all genes; “pvalue”: p-value reported by the enricher function from the R package "clusterProfiler"; “p.adjust”: Benjamini and Hochberg adjusted p-values; “qvalue”: Expected pFDR; “geneID”: Upregulated input genes present in a given GO term; “Count”: Number of upregulated input genes present in a given GO term.

Supplementary Table 5. Comprehensive evaluation of gene signatures expression trends as a function of tumour density, related to Extended Data Fig. 2g-o
Enrichment trends of a series of GO categories gene signatures as a function of binned tumour densities were tested using a Mann-Kendall Trend Test (See Method for detailed explanation). Eight clusters of “S” values generated by the “mk.test” function from the R package “trend” are reported for 10 PDX tumours. Positive/Negative “S” are signs of increasing/decreasing trends respectively.

Supplementary Table 6. GO terms labelling key, related to Figure 2c, e, g and Extended Data Figure 2q
Labels shown on the figures are reported on the left column and corresponding GO term names on the right column.

Supplementary Table 7. Human white matter markers derived from the *Ravi et al* study, related to Fig. 2g

Marker genes derived in this study and used to identify white matter-rich ST spots in human datasets (see methods).

Supplementary Table 8. Processing statistics for single-cell RNA sequencing data, related to Fig. 5e-h and Extended Data Fig. 9

Output of Cell Ranger 7.0.1 for five 10x Genomics Chromium Next GEM datasets from npp tumour from two WT (WB_1, WB_2) and three *Sarm1*$^{-/-}$ mice (SB_1, SB_2, SB_3). Columns 6 to 18 are fractions.

Supplementary Table 9. Meta data for single-cell RNA-seq experiments, related to Fig. 5e-h and Extended Data Fig. 9

Annotation of all cells reported in this study. "LABELS": Cell labels manually curated in this study; "prediction from other studies": Predicted labels used for tumour cells assignment (see methods); "tdTomato": Number of tdTomato UMIs used for tumour cells assignment; "copyKat": Ploidy as assessed using the copyKat package and used for tumour cells assignment (see methods).
Cell type labels key:
Cells from Antunes study: dendritic cell (DC1, DC2, DC3 and DC4), Tumor-associated macrophages (TAM 1 and TAM 2), Monocytes, proliferate TAM (prol. TAM) and Mast cells (Mast);
Cells from Yeo study: astrocyte (ASC), Ciliated cells, dendritic cell (DC), endothelial cell (EC), macrophage (MAC), microglia (MG), Mast, Monocytes, Neuroendocrine, neutrophil (NEUT), oligodendrocyte (OLG), T cells, TumoriCre-Differentiatedlike1, TumoriCre-Differentiatedlike2, TumoriCre-ProlifStemlike, TumoriCre-Stemlike1 and TumoriCre-Stemlike2
Cells from Ximerakis study: arachnoid barrier (ABC), astrocyte (ASC), dendritic cell (DC), endothelial cell (EC), hemoglobin expressing vascular (Hb_VC), immature neurons (ImmN), macrophage (MAC), microglia (MG), Monocytes, neurons (mNEUR), Neuroendocrine, neutrophil (NEUT), neuronal restricted precursors (NRP), neural stem cell (NSC), oligodendrocyte (OLG), oligodendrocyte progenitor cells (OPC), pericytes (PC), vascular and leptomeningeal (VLMC) and vascular smooth muscle (VSMC).
Cells from Kalamakis study: transient amplifying progenitors (TAP), active neural stem cells (aNSC1, aNSC2), neuroblasts (NB) and astrocyte (ASC, originally labelled as quiescence neural stem cells (qNSC1 and qNSC2))

Supplementary Table 10. Gene enrichment analysis of cell type markers in tumour and TME cells from WT and *Sarm1*$^{-/-}$ mice, related to Fig. 5e-f

Gene enrichment analysis performed on cell types defined from scRNA-seq data (see methods). "GeneRatio": Ratio of upregulated input genes present in a given GO term over the total number of upregulated input genes; "BgRatio": Ratio of all genes annotated in a given GO term over all genes; "pvalue": p-value reported by the enricher function from the R package "clusterProfiler"; "p.adjust": Benjamini and Hochberg adjusted p-values; "qvalue": Expected pFDR; "geneID": Upregulated input genes present in a given GO term; "Count": Number of upregulated input genes present in a given GO term. "cell type": Labels manually curated in this study.
