# Supplementary material for: Axonal injury is a targetable driver of glioblastoma progression
Source: Nature. 2025 Aug 20;646(8084):452–61. doi: 10.1038/s41586-025-09411-2 (PMC12507684; doi:10.1038/s41586-025-09411-2)
Supplement: Supplementary file 2 — Reporting Summary [file 41586_2025_9411_MOESM2_ESM.pdf]

nature portfolio

Corresponding author(s): Simona Parrinello; Ciaran Hill

Last updated by author(s): Jun 12, 2025

Reporting Summary

Nature Portfolio wishes to improve the reproducibility of the work that we publish. This form provides structure for consistency and transparency in reporting. For further information on Nature Portfolio policies, see our Editorial Policies and the Editorial Policy Checklist.

Statistics

For all statistical analyses, confirm that the following items are present in the figure legend, table legend, main text, or Methods section.

n/a	Confirmed	
☐	☒	The exact sample size (n) for each experimental group/condition, given as a discrete number and unit of measurement
☐	☒	A statement on whether measurements were taken from distinct samples or whether the same sample was measured repeatedly
☐	☒	The statistical test(s) used AND whether they are one- or two-sided<br>*Only common tests should be described solely by name; describe more complex techniques in the Methods section.*
☒	☐	A description of all covariates tested
☐	☒	A description of any assumptions or corrections, such as tests of normality and adjustment for multiple comparisons
☐	☒	A full description of the statistical parameters including central tendency (e.g. means) or other basic estimates (e.g. regression coefficient) AND variation (e.g. standard deviation) or associated estimates of uncertainty (e.g. confidence intervals)
☐	☒	For null hypothesis testing, the test statistic (e.g. F, t, r) with confidence intervals, effect sizes, degrees of freedom and P value noted<br>*Give P values as exact values whenever suitable.*
☒	☐	For Bayesian analysis, information on the choice of priors and Markov chain Monte Carlo settings
☒	☐	For hierarchical and complex designs, identification of the appropriate level for tests and full reporting of outcomes
☐	☒	Estimates of effect sizes (e.g. Cohen's d, Pearson's r), indicating how they were calculated

Our web collection on statistics for biologists contains articles on many of the points above.

Software and code

Policy information about availability of computer code

Data collection	Confocal images were acquired using Zeiss Zen black 2.1 and 3i SlideBook (2023). Sequencing data were collected on a NovaSeq at Novogene, Cambridge, UK. Flow data were aquired using BD FACSymphony™, FACS DIVA version 9.1.
Data analysis	Preprocessing of scRNA-seq data: 10x Genomics Cell Ranger 7.0.1; Preprocessing of Visium data: 10x Genomics Space Ranger 2.0.1.<br>R version 4.3.2 (2023-10-31): R packages including bayNorm (1.24.0), BayesSpace (1.17.0), Seurat (5.2.1), randomForest (4.7-1.2), presto (1.0.0), clusterProfiler (4.14.6), ComplexHeatmap (2.22.0), Harmony (1.2.3), DESeq2 (1.46.0), liana (0.1.14), copykat (1.1.0), ggplot2 (3.5.1), trend (1.1.6), AUCell (1.28.0) and Rsamtools (2.22.0) were used to analyze the data. Python 3.9.16: Python packages including squidpy (1.6.3), cell2location (0.1.3) and scanpy (1.11.0) were used to analyze the data.<br>Imaris v10.1.0<br>FlowJo v10.8.1<br>Fiji ImageJ v1.54f<br>GraphPad Prism v10<br>MyelTracer v1.3.1<br><br>Original code as well as the input data used to generate the main analyses of the paper are publicly available at: https://doi.org/10.5281/zenodo.15608353 and https://github.com/WT215/Axonal-Injury

For manuscripts utilizing custom algorithms or software that are central to the research but not yet described in published literature, software must be made available to editors and reviewers. We strongly encourage code deposition in a community repository (e.g. GitHub). See the Nature Portfolio guidelines for submitting code & software for further information.

Data

Policy information about availability of data

All manuscripts must include a data availability statement. This statement should provide the following information, where applicable:

- Accession codes, unique identifiers, or web links for publicly available datasets
- A description of any restrictions on data availability
- For clinical datasets or third party data, please ensure that the statement adheres to our policy

Reference genomes GRCh38-2020-A (human) and mm10-2020-A (mouse) were downloaded from https://www.10xgenomics.com/support/cn/software/space-ranger/downloads#reference-downloads.
Sequencing data generated in this study has been deposited in GEO with the following accession codes GSE268312 for ST data; GSE268298 for scRNA-seq data. The scRNA-seq dataset from Ximerakis et al. is available at GSE129788 (https://www.ncbi.nlm.nih.gov/geo/query/acc.cgi?acc=GSE129788), cells from young mice were used (2–3-month-old). The scRNA-seq dataset from Antunes et al. was obtained at GSE163120 (https://www.ncbi.nlm.nih.gov/geo/query/acc.cgi?acc=GSE163120), cells from WT mice were used. The scRNA-seq dataset from Kalamakis et al. is available at GSE115626 (https://www.ncbi.nlm.nih.gov/geo/query/acc.cgi?acc=GSE115626), cells from young mice were used (2-month-old). The scRNA-seq dataset from Yeo et al. is available at GSE195848 (https://www.ncbi.nlm.nih.gov/geo/query/acc.cgi?acc=GSE195848). The human ST dataset from Ravi et al. was obtained from https://datadryad.org/stash/dataset/doi:10.5061/dryad.h70rxwdmj. Source data are provided for this paper. The human Cosmx dataset from Moffet et al. was obtained from https://data.mendeley.com/datasets/wc8tmdmsxm/3.

Research involving human participants, their data, or biological material

Policy information about studies with human participants or human data. See also policy information about sex, gender (identity/presentation), and sexual orientation and race, ethnicity and racism.

Reporting on sex and gender	N/A
Reporting on race, ethnicity, or other socially relevant groupings	N/A
Population characteristics	N/A
Recruitment	The participants will be identified by their treating physician in a neuro-oncology clinic or when they are admitted to hospital. If the patient has consented to surgery to remove a presumed glioma- and the treating physicians feels it is appropriate- they will ask the patient if they would like information on a research project investigating the growth and spread of brain tumours. If they are interested then the patient will be provided with the Patient Information Sheet and a copy of the Consent form. Their contact details will be passed to the Co-Investigator (Dr Ciaran Hill) who will contact them to explain the project and answer any questions. They will be given a minimum of 24 hours to consider before consenting.
Ethics oversight	Study title: Mechanisms of Glioma Invasion in the Human Brain approved by HRA and Health and Care Researh Wales REC Ref: 20/WA/0251 IRAS project ID: 280746

Note that full information on the approval of the study protocol must also be provided in the manuscript.

Field-specific reporting

Please select the one below that is the best fit for your research. If you are not sure, read the appropriate sections before making your selection.

☒ Life sciences ☐ Behavioural & social sciences ☐ Ecological, evolutionary & environmental sciences

For a reference copy of the document with all sections, see nature.com/documents/nr-reporting-summary-flat.pdf

Life sciences study design

All studies must disclose on these points even when the disclosure is negative.

Sample size	Sample size for all experiments was based on previously published studies and from previous experience using the same models. No statistics were used to predetermine sample size. (Brooks et al. 2021; Krusche et al. 2016; Garcia-Diaz et al., 2023)
Data exclusions	A minority of tumours developed intraventricularly and were excluded as per predefined criteria.
Replication	Replicates were used in all experiments as noted in the text. All experiments were repeated at least three times with reproducible results.
Randomization	For xenogfraft experiments cohorts of 5 mice recieved the same cell line injections but were randomly assigned id and collected based on IVIS signal and manifestation of disease progression in line with home office end points. Collected brains were randomly assigned to spatial transcriptomic experiments apart from both control NOD.CB17-Prkdcscid/NCrCrl mice which were used for baseline transcriptomic analysis.

For the majority of experiments including the time course experiments (Figure 1 and Extended Data Figure 1, Figure 3 and Extended Data Figure 3, scRNA sequencing experiments Figure 5 and Extended Data Figure 9), mice were randomly collected for processing.
For axonal transection injury experiments (Figure 4 and Extended Data Figure 5,6) male and female mice were randomised separately into Sham and injury groups. For AAV intratumoural injection experiment (Extended Data Figure 6) male and female mice were randomised separately into GFP and SarmDN groups.

Blinding

For time course experiment (Figure 1 and Extended Data Figure 1) blinding was not possible due to the obvious presence of tumour cells. For injury experiments (Figure 4 and Extended Data Figure 5,6) it was not possible to blind the analysis as it was obvious where the site of injury was. Analysis of AAV experiments (Extended Data Figure 6) and vascular phenotypes (Extended Data Figure 8 d-n) was performed blind. All quantifications were automated where possible to remove operator bias.

Reporting for specific materials, systems and methods

We require information from authors about some types of materials, experimental systems and methods used in many studies. Here, indicate whether each material, system or method listed is relevant to your study. If you are not sure if a list item applies to your research, read the appropriate section before selecting a response.

Materials & experimental systems

n/a	Involved in the study
☐	☒ Antibodies
☐	☒ Eukaryotic cell lines
☒	☐ Palaeontology and archaeology
☐	☒ Animals and other organisms
☒	☐ Clinical data
☒	☐ Dual use research of concern
☒	☐ Plants

Methods

n/a	Involved in the study
☒	☐ ChIP-seq
☐	☒ Flow cytometry
☒	☐ MRI-based neuroimaging

Antibodies

Antibodies used

rabbit anti-Ki67 (1:250; Abcam, ab16667); goat anti-GFAP (1:1,000; Abcam, ab53554); rat anti-CD68 (1:500; Abcam, ab53444); rabbit anti-Iba1 (1:1,000; Wako, 019–19741); L0159), mouse anti-neurofilament H (1:1000 Enzo ENZ-ABS219-0100); mouse anti-myelin basic protein (1:1000 Covance SMI-99); mouse anti SMI32 (1:1000, Enzo, ENZ-ABS219-0010) chicken anti-GFP (1:1000, Abcam ab13970), rabbit anti-RFP (1:1000, ABIN129578), rabbit anti-pMLC2 (1:100, Cell signalling 3671), mouse anti-phospho-Tau S202/T205 (1:500, a kind gift from G.Schiavo), mouse anti-TDP-43 (1:500, Abcam ab104223), rabbit anti-TOMM20 (1:1000, ab186735), mouse anti-Amyloid beta (1:100 Merk, MAB348A4), rabbit anti-laminin (1:500, Sigma, L9393), goat anti-CD31 (1:100, BioTechne, AF3628), rat anti-PdgfrB (1:200, kind gift from I.Kim), donkey anti-mouse IgG 488 (1:500, ThermoFisher A21202), rat anti-LY6G-BUV563 (1:100, Clone IA8, BD, 612921); rat anti-CD11b-BUV661 (1:400, Clone M1/70, BD, 612977); rat anti-MHC Class II-BB700 (1:800, Clone M5/114.15.2, BD, 746197); mouse anti-CD45-BUV805 (1:400, Clone 30-F11, BD, 748370), mouse anti-CD64-BV421 (1:100, Clone X54-5/7.1, Biolegend, 139309), mouse anti-CX3CR1-BV510 (1:400 Clone SA011f11, Biolegend 149025); rat anti-LY6C-BV605 (1:200, Clone AL-21, BD 563011); rat anti-CD19-BV650 (1:50, Clone ID3, BD 563235); hamster anti-CD11C-BV785 (1:100, Clone N418, Biolegend 117336); rat anti-CD49d-APC (1:200, Clone R1-2, Biolegend 103622); rat anti-F4/80-AF700 (1:100, Clone BM8, Biolegend 123130); mouse anti-Ki67-BUV395 (1:100, Clone B56, BD 564071); rat anti-CD3-BUV737 (1:300, Clone 17A2, BD564380); rat anti-CD206-AF488 (1:100, Clone C068C2, Biolegend 141710); donkey anti-rabbit Alexa Fluor 647 (1:1000 Thermo Fisher A-31573).

Validation

All antibodies have been validated in the literature and/or had validation data supplied by the manufacturer. Further validation was performed to confirm that each antibody produced the expected cellular patterns.

rabbit anti-Ki67 (1:250; Abcam, ab16667), 3,277 citations. Advanced validation on manufacturers website.
goat anti-GFAP (1:1,000; Abcam, ab53554), >471 citations. Validated by positive signal in cells known to express it.
rat anti-CD68 (1:500; Abcam, ab53444), > 360 citations. Validation on manufacturers website.
rabbit anti-Iba1 (1:1,000; Wako, 019–19741), >587 citations. Validation on manufacturers website.
mouse anti-neurofilament H (1:1000 Enzo ENZ-ABS219-0100), =1 citation. Validated in house by positive signal in cells known to express it.
mouse anti-myelin basic protein (1:1000 Covance SMI-99), >27 citations. Validation on manufacturers website.
mouse anti SMI32 (1:1000, Enzo, ENZ-ABS219-0010), 1 citation. Validated in house by positive signal in cells known to express it.
chicken anti-GFP (1:1000, Abcam ab13970), >3,612 citations. Validation on manufacturers website.
rabbit anti-RFP (1:1000, ABIN129578), >300 citations. Validation on manufacturers website.
rabbit anti-pMLC2 (1:100, Cell signalling 3671), >795 citations. Validation on manufacturers website.
mouse anti-phospho-Tau S202/T205 (1:500, a kind gift from G.Schiavo), >2 citations. Validated in house by positive signal in cells known to express it.
mouse anti-TDP-43 (1:500, Abcam ab104223), >18 citations. Validation on manufacturers website.
rabbit anti-TOMM20 (1:1000, ab186735), >161 citations. Validation on manufacturers website.
mouse anti-Amyloid beta (1:100 Merk, MAB348A4), 5 citations. Validation on manufacturers website.
rabbit anti-laminin (1:500, Sigma, L9393), >2,125 citations. Validation on manufacturers website.
goat anti-CD31 (1:100, BioTechne, AF3628), >915 citations. Validation on manufacturers website.
rat anti-PdgfrB (1:200, kind gift from I.Kim https://www.nature.com/articles/s12276-023-00939-9) Validated in house by positive signal in cells known to express it.
donkey anti-mouse IgG 488 (1:500, ThermoFisher A21202), >5,645 citations. Validation on manufacturers website.
rat anti-LY6G-BUV563 (1:100, Clone IA8, BD, 612921), >14 citations. Validation on manufacturers website.

rat anti-CD11b-BUV661 (1:400, Clone M1/70, BD, 612977), >4 citations. Validation on manufacturers website.
rat anti-MHC Class II-BB700 (1:800, Clone M5/114.15.2, BD, 746197), > 7 citations. Validation on manufacturers website.
mouse anti-CD45-BUV805 (1:400, Clone 30-F11, BD, 748370), >13 citations. Validation on manufacturers website.
mouse anti-CD64-BV421 (1:100, Clone X54-5/7.1, Biolegend, 139309), >69 citations. Validation on manufacturers website.
mouse anti-CX3CR1-BV510 (1:400 Clone SA011f11, Biolegend 149025), >5 citations. Validation on manufacturers website.
rat anti-LY6C-BV605 (1:200, Clone AL-21, BD 563011), >35 citations. Validation on manufacturers website.
rat anti-CD19-BV650 (1:50, Clone ID3, BD 563235), >15 citations. Validation on manufacturers website.
hamster anti-CD11C-BV785 (1:100, Clone N418, Biolegend 117336), >41 citations. Validation on manufacturers website.
rat anti-CD49d-APC (1:200, Clone R1-2, Biolegend 103622), >4 citations. Validation on manufacturers website.
rat anti-F4/80-AF700 (1:100, Clone BM8, Biolegend 123130), >41 citations. Validation on manufacturers website.
mouse anti-Ki67-BUV395 (1:100, Clone B56, BD 564071), >20 citations. Validation on manufacturers website.
rat anti-CD3-BUV737 (1:300, Clone 17A2, BD 564380), >17 citations. Validation on manufacturers website.
rat anti-CD206-AF488 (1:100, Clone C068C2, Biolegend 141710), >38 citations. Validation on manufacturers website.
donkey anti-rabbit Alexa Fluor 647 (1:1000 Thermo Fisher A-31573). Validation on manufacturers website.

Eukaryotic cell lines

Policy information about cell lines and Sex and Gender in Research

Cell line source(s): Human cell lines were acquired from the CRUK glioma cellular genetics resource or prepared in house from anonymised patient material

Authentication: *Describe the authentication procedures for each cell line used OR declare that none of the cell lines used were authenticated.*

Mycoplasma contamination: All cell lines were tested and were mycoplasma free

Commonly misidentified lines (See ICLAC register): *Name any commonly misidentified cell lines used in the study and provide a rationale for their use.*

Animals and other research organisms

Policy information about studies involving animals; ARRIVE guidelines recommended for reporting animal research, and Sex and Gender in Research

Laboratory animals: C57BL/6NCrl (Charles River) RRID:MGI:2683688; B6.Cg-Tg(Thy1-YFP)HJrs/J RRID:IMSR_JAX:003709; sterile alpha and TIR Motif1-/- Sarm1tm1Aidi (RRID 018069; a gift from M.Coleman); Sarm1em1.1Tftc and Sarm1 wild-type (Doran et al, 2021); NOD.CB17-Prkdcscid/NCrCrl RRID:IMSR_CRL:394; Apptm3.1Tcs (RRID 5637817, a gift from S.Hong) and rTg4510 (024854, a gift from G. Schiavo). Wild-type or Sarm1-/- mice were sacrificed either at terminal disease (median 125 days wild-type; 148 days Sarm1-/-) or at specified time points (early: less than 8 weeks; intermediate: 8-12 weeks; late: 12-15 weeks). NOD.CB17-Prkdcscid/NCrCrl xenograft mice were sacrificed at the half-way point of tumour development, or when the tumours were terminal which was 34-70 days early; 56-190 days for terminal. NOD.CB17-Prkdcscid/NCrCrl control mice for spatial transcriptomics were sacrificed at 15 weeks.

Wild animals: No wild animals were used in this study.

Reporting on sex: Both male and female mice were used.

Field-collected samples: No field collected samples were used in this study.

Ethics oversight: UK Home Office Licence number PP5770663 approved by UCL AWERB approved

Note that full information on the approval of the study protocol must also be provided in the manuscript.

Plants

Seed stocks: N/A

Novel plant genotypes: *Describe the methods by which all novel plant genotypes were produced. This includes those generated by transgenic approaches, gene editing, chemical/radiation-based mutagenesis and hybridization. For transgenic lines, describe the transformation method, the number of independent lines analyzed and the generation upon which experiments were performed. For gene-edited lines, describe the editor used, the endogenous sequence targeted for editing, the targeting guide RNA sequence (if applicable) and how the editor was applied.*

Authentication: *Describe any authentication procedures for each seed stock used or novel genotype generated. Describe any experiments used to assess the effect of a mutation and, where applicable, how potential secondary effects (e.g. second site T-DNA insertions, mosiacism, off-target gene editing) were examined.*

Flow Cytometry

Plots

Confirm that:

☒ The axis labels state the marker and fluorochrome used (e.g. CD4-FITC).

☒ The axis scales are clearly visible. Include numbers along axes only for bottom left plot of group (a 'group' is an analysis of identical markers).

☒ All plots are contour plots with outliers or pseudocolor plots.

☒ A numerical value for number of cells or percentage (with statistics) is provided.

Methodology

Sample preparation	Brains were collected into ice-cold HBSS media and dissected into 1mm coronal sections using a brain matrix (World Precision Instruments, RBMS200C). Tumour regions were dissected out and mechanically dissociated into small pieces, followed by enzymatic dissociation using Liberase TL (Roche, 05401119001) supplemented with DNAse I (Merck, 11284932001) for 30 min at 37oC. Following addition of EDTA to stop the enzymatic reaction, cells were washed with PBS and filtered through a 70mm cell strainer (Falcon, 352350) to remove large debris. Samples were blocked on ice for 20 min (BioXCell blocking buffer; BE0307) prior to incubation in antibodies and fixable viability dye eFluor780 (eBioscience, 65-0865-18, 1:1000) at 4oC for 20 min. To detect immune cells within the tumour population the following antibodies were used rat anti-LY6G-BUV563 (1:100, Clone IA8, BD, 612921), rat anti-CD11b-BUV661 (1:400, Clone M1/70, BD, 612977), rat anti-MHC Class II-BB700 (1:800, Clone M5/114.15.2, BD, 746197), mouse anti-CD45-BUV805 (1:400, Clone 30-F11, BD, 748370), mouse anti-CD64-BV421 (1:100, Clone X54-5/7.1, Biolegend, 139309), mouse anti-CX3CR1-BV510 (1:400 Clone SA011f11, Biolegend 139309), rat anti-LY6C-BV605 (1:200, Clone AL-21, BD 563011), rat anti-CD19-BV650 (1:50, Clone ID3, BD 563235), hamster anti-CD11C-BV785 (1:100, Clone N418, Biolegend 117336), rat anti-CD49d-APC (1:200, Clone R1-2, Biolegend 103622), rat anti-F4/80-AF700 (1:100, Clone BM8, Biolegend 123130), mouse anti-Ki67-BUV395 (1:100, Clone B56, BD 564071), rat anti-CD3-BUV737 (1:300, Clone 17A2, BD564380), rat anti-CD206-AF488 (1:100, Clone C068C2, Biolegend 141710). Data was analyzed using Flowjo (v10.7.1; RRID:SCR_008520). Data was compensated, fluorescence minus one controls were generated, and only viable singlets were used for downstream analysis.
Instrument	BD FACSymphony (LSRFortessa X-50) flow cytometer (Reference 66096451, model NA)
Software	BD FACSDiva™ Software - version 9.1<br>BD FlowJo™ Software - version 10.8.1
Cell population abundance	N/A (no sorting experiments were used in this paper)
Gating strategy	All cells were first gated in FSC/SSC according to cell size and granularity. This population was then gated in FSC-A/FSC-H to contain only single cells. Next, single cells were gated based on the viability dye, and live cells (negative population) were used for further cell type identification. CD45+ cells were identified in CD45/CD11b. This immune cell population was then gated in CD45/CD11b again to separate myeloid population (CD11b high) and lymphocytic population (CD11b low). The myeloid cells were then further gated in LY6C/LY6G for the double negative population corresponding to tumour associated microglia/macrophages (TAMs). Finally, this population was gated in CD45/CD49d to separate microglia and peripherally derived macrophages (CD45 and CD49d high).

☒ Tick this box to confirm that a figure exemplifying the gating strategy is provided in the Supplementary Information.
